# Overlapping and distinct roles of CDPK family members in the pre-erythrocytic stages of the rodent malaria parasite, *Plasmodium berghei*

**DOI:** 10.1371/journal.ppat.1008131

**Published:** 2020-08-31

**Authors:** Kavitha Govindasamy, Purnima Bhanot

**Affiliations:** Rutgers New Jersey Medical School, Department of Microbiology, Biochemistry and Molecular Genetics, Newark, New Jersey, United States of America; Francis Crick Institute, UNITED KINGDOM

## Abstract

Invasion of hepatocytes by *Plasmodium* sporozoites initiates the pre-erythrocytic step of a malaria infection. Subsequent development of the parasite within hepatocytes and exit from them is essential for starting the disease-causing erythrocytic cycle. Identification of signaling pathways that operate in pre-erythrocytic stages provides insight into a critical step of infection and potential targets for chemoprotection from malaria. We demonstrate that *P*. *berghei* homologs of Calcium Dependent Protein Kinase 1 (CDPK1), CDPK4 and CDPK5 play overlapping but distinct roles in sporozoite invasion and parasite egress from hepatocytes. All three kinases are expressed in sporozoites. All three are required for optimal motility of sporozoites and consequently their invasion of hepatocytes. Increased cGMP can compensate for the functional loss of CDPK1 and CDPK5 during sporozoite invasion but cannot overcome loss of CDPK4. CDPK1 and CDPK5 expression is downregulated after sporozoite invasion. CDPK5 reappears in a subset of late stage liver stages and is present in all merosomes. Chemical inhibition of CDPK4 and depletion of CDPK5 in liver stages implicate these kinases in the formation and/or release of merosomes from mature liver stages. Furthermore, depletion of CDPK5 in merosomes significantly delays initiation of the erythrocytic cycle without affecting infectivity of hepatic merozoites. These data suggest that CDPK5 may be required for the rupture of merosomes. Our work provides evidence that sporozoite invasion requires CDPK1 and CDPK5, and suggests that CDPK5 participates in the release of hepatic merozoites.

## Author summary

The malaria-parasite *Plasmodium* begins its mammalian cycle by infecting hepatocytes in the liver. A single parasite differentiates into tens of thousands of hepatic merozoites which exit the host cell in vesicles called merosomes. Hepatic merozoites initiate the first round of erythrocytic infection that eventually causes disease. We show that optimal invasion of liver cells by *Plasmodium* requires the action of three closely-related parasite kinases, CDPK1, 4 and 5. Loss of any of the three enzymes in the parasite significantly reduces infection of liver cells. Furthermore, CDPK5 is likely required for release of hepatic merozoites from merosomes and therefore for initiation of the erythrocytic cycle. A better understanding of how these kinases function could lead to drugs that prevent malaria.

## Introduction

Parasite genomes are generally small in size with a great deal of functional optimization. The presence of gene families suggests that in some cases, structurally related proteins play redundant or complementary functions. One such gene family in the malaria-causing parasite, *Plasmodium* encodes Calcium Dependent Protein Kinases (CDPKs). In addition to Apicomplexan parasites, CDPKs are found in plants, protists, green alga and oomycetes [1]. CDPKs are serine-threonine kinases that are activated by the direct binding of Ca^2+^ to EF-hand domains in their regulatory region. Their ability to directly bind Ca^2+^ enables CDPKs to act both as sensors and effectors of intracellular Ca^2+^.

The human-infective species, *P*. *falciparum* encodes at least 7 CDPK members [2]. The rodent-infective species, *P*. *berghei* encodes orthologs of all *P*. *falciparum* CDPKs except for *P*. *falciparum* CDPK2. The function of different CDPKs has been extensively examined in the asexual cycle in erythrocytes and during parasite transmission to and development within mosquitoes. These studies revealed distinct and overlapping roles for different CDPK members during merozoite invasion and egress, gametogenesis and ookinete invasion. In the asexual cycle, CDPK5 is required for merozoite egress [3, 4], while CDPK1 and CDPK4 play non-essential roles in merozoite invasion [5, 6]. Invasion by *P*. *berghei* merozoites is reduced when CDPK4 is lost in the background of reduced cGMP-dependent protein kinase (PKG) function but loss of CDPK4 alone does not affect asexual growth of *P*. *berghei* or of *P*. *falciparum* [6]. Similarly, in the background of simultaneous decrease in PKG and CDPK4 functions, CDPK1’s loss decreases merozoite invasion [6, 7] but its individual loss is tolerated in the asexual cycle [6, 8, 9]. CDPK2, CDPK3 and CDPK6 do not appear to play significant roles in the asexual cycle [10–13]. In sexual stages within the mosquito midgut, CDPK1, CDPK2 and CDPK4 are essential for male gametogenesis with CDPK4 regulating at least 3 distinct cell cycle events [13–18]. Ookinete motility requires CDPK3 [10, 11] as it regulates the secretion of adhesins required for motility. CDPK1 and CDPK4 also contribute to ookinete motility: simultaneous loss of both reduced ookinete speed *in vitro* [6]. These studies of CDPK family members in the mosquito transmissive stages have provided biological validation for targeting *P*. *falciparum* CDPK1 and CDPK4 as a way to chemically block parasite transmission from mammalian host to mosquitoes.

Sporozoites and liver stages express several CDPKs [8, 12, 19] but, in contrast to our knowledge in asexual and sexual cycles, little is known of CDPKs’ functions in pre-erythrocytic stages. CDPK4 and CDPK6 function during invasion by *P*. *berghei* sporozoites since their loss reduces the percentage of sporozoites that enter hepatocytes [12, 19]. However, deletion of CDPK1 in sporozoites did not significantly impair parasite invasion of or egress from hepatocytes [8]. These results suggested that other CDPKs could play a compensatory and complementary role during sporozoite invasion and parasite exit from hepatocytes.

Here we examine the role of CDPK1, CDPK4 and CDPK5 in sporozoite motility, and traversal through, invasion of and egress from infected hepatocytes, using conditional protein depletion in the rodent model, *P*. *berghei*. *P*. *berghei* offers the distinct advantage of an experimentally accessible pre-erythrocytic cycle. We demonstrate that in *P*. *berghei* sporozoites, CDPK1 (PBANKA_0314200), CDPK4 (PBANKA_0615200) and CDPK5 (PBANKA_1351500) are individually required for motility. The loss of each kinase decreases sporozoite motility and consequently significantly reduces their invasion of hepatocytes. CDPK5 is required for parasite egress from hepatocytes and for release of hepatic merozoites from merosomes. Our study demonstrates the roles of CDPK1 and CDPK5 in sporozoite invasion. Furthermore, it provides evidence that CDPK5 could participate in the regulated release of hepatic merozoites from merosomes.

## Results

### CDPK1, CDPK4 and CDPK5 have dynamic expression patterns in pre-erythrocytic stages

Since CDPK1, 4, 5 have essential roles in the asexual and sexual cycles, genetic analyses of their functions in pre-erythrocytic stages requires conditional mutagenesis. Existing methods face several drawbacks. Excision of DNA in developing sporozoites, through the use of the Flp-FRT system [20], may not lead to significant protein depletion if the targeted protein has a long half-life [19, 21], or if DNA excision is initiated after most of the mRNA has been transcribed. Promoter-swaps require knowledge of promoters that are active exclusively during asexual stages and sporozoite development. Systems in which target-protein stability is conditioned on the constant presence of a small molecule in culture [22–24] cannot ensure protein stability during the sexual cycle and sporozoite development of the parasite that occur *in vivo*. Therefore, we adapted a method, previously described in *P*. *berghei* asexual stages and ookinetes, in which the target protein is degraded upon the addition of the plant hormone, auxin (Indole-3-acetic acid (IAA)) [25, 26]. The target protein is tagged with an Auxin-induced degron (AID) in a marker-free transgenic *P*. *berghei* line that expresses the auxin receptor from *Oryza sativa*, OsTIR1under the control of the *hsp70* promoter and the *p28* 3’ UTR. [27]. The tag targets the protein for rapid and reversible degradation, via the proteasome, in the presence of auxin. In *S*. *cerevisiae* and mammalian cells, the AID system has been used to target proteins localized to the cytoplasm, nucleus, mitochondria and ER [25, 26]. We constructed parasite lines in which CDPK4 or CDPK5 were fused with an AID domain and an HA_2x_ epitope (S1A and S1B Fig). These two parasite lines, CDPK4-aid-HA and CDPK5-aid-HA, and a previously reported CDPK1-aid-HA line [27] were transmitted to mosquitoes for recovery of sporozoites from salivary glands.

A qualitative microscopic examination of midguts revealed fewer oocysts in mosquitoes infected with CDPK1-aid-HA or CDPK4-aid-HA parasites compared to mosquitoes infected with an isogenic control line that expresses OsTIR1 (Ostir1). Oocyst numbers in mosquitoes infected with CDPK5-aid-HA were equivalent to control. Since genomic deletions of CDPK1 and CDPK4 abolish male gametogenesis [15, 16], the decrease in oocyst numbers in CDPK1-aid-HA and CDPK4-aid-HA lines suggest that the AID-HA_2x_ domain interferes with protein activity and therefore, partially impairs gametogenesis in these lines. Consistent with reduced midgut infection by CDPK1-aid-HA and CDPK4-aid-HA, the average number of salivary gland sporozoites was significantly decreased in mosquitoes infected with CDPK1-aid-HA and CDPK4-aid-HA, relative to Ostir1-infected mosquitoes (S1C Fig). The number of salivary gland sporozoites in mosquitoes infected with CDPK5-aid-HA was similar to control (S1C Fig). Despite reduced numbers of CDPK1-aid-HA, we obtained sufficient parasites to conduct our analyses. Due to the low numbers of CDPK4-aid-HA sporozoites, their analysis was more limited. As shown by subsequent results, sporozoites derived from AID-tagged lines exhibited phenotypes within the range observed for control sporozoites. Therefore, if IAA-dependent depletion of a protein alters a cellular phenotype, it can be inferred that the protein has a role in the process.

**Fig 1:**
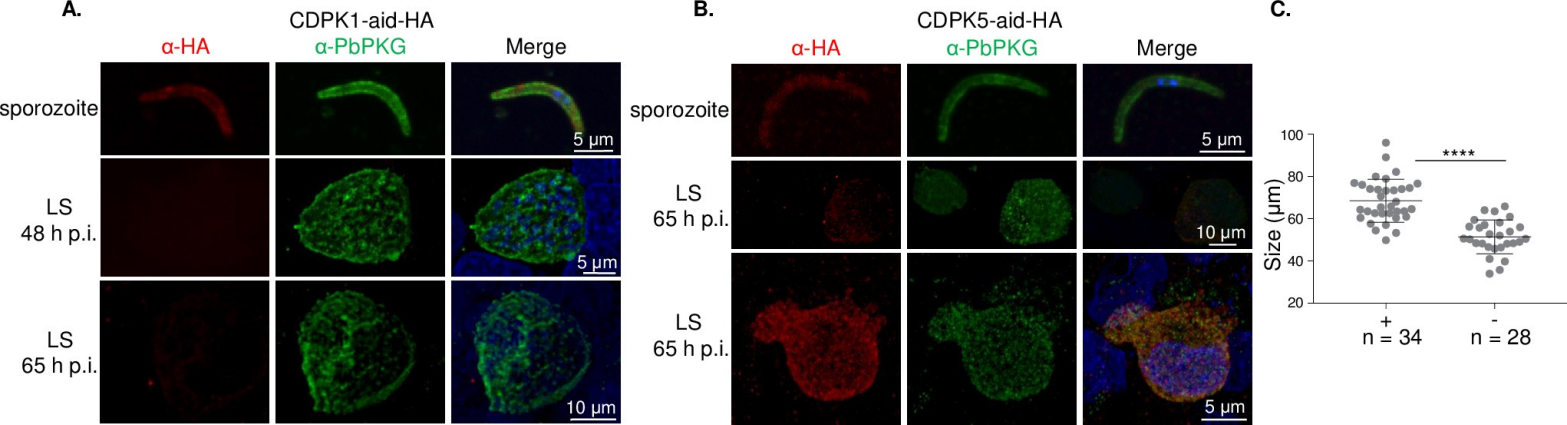
Spatial and temporal localization of CDPK1 and CDPK5 in *P*. *berghei* pre-erythrocytic stages. **A)** Representative images of CDPK1 localization in pre-erythrocytic stages of CDPK1-aid-HA parasites determined using an anti-HA antibody. CDPK1 was detected in sporozoites but not in liver stages. **B)** Representative images of CDPK5 localization in pre-erythrocytic stages of CDPK5-aid-HA parasites determined using an anti-HA antibody. CDPK5 was detected in all sporozoites and in a subset of mature liver stages (65 h p.i.). **C)** Diameter of individual CDPK5-aid-HA liver stages (μM ± SEM), that either displayed staining with anti-HA antibody at 65 h p.i. (±) or not (-) was determined. Data were analyzed using an unpaired *t*-test, **** *P* value < 0.0001.

**Fig 2:**
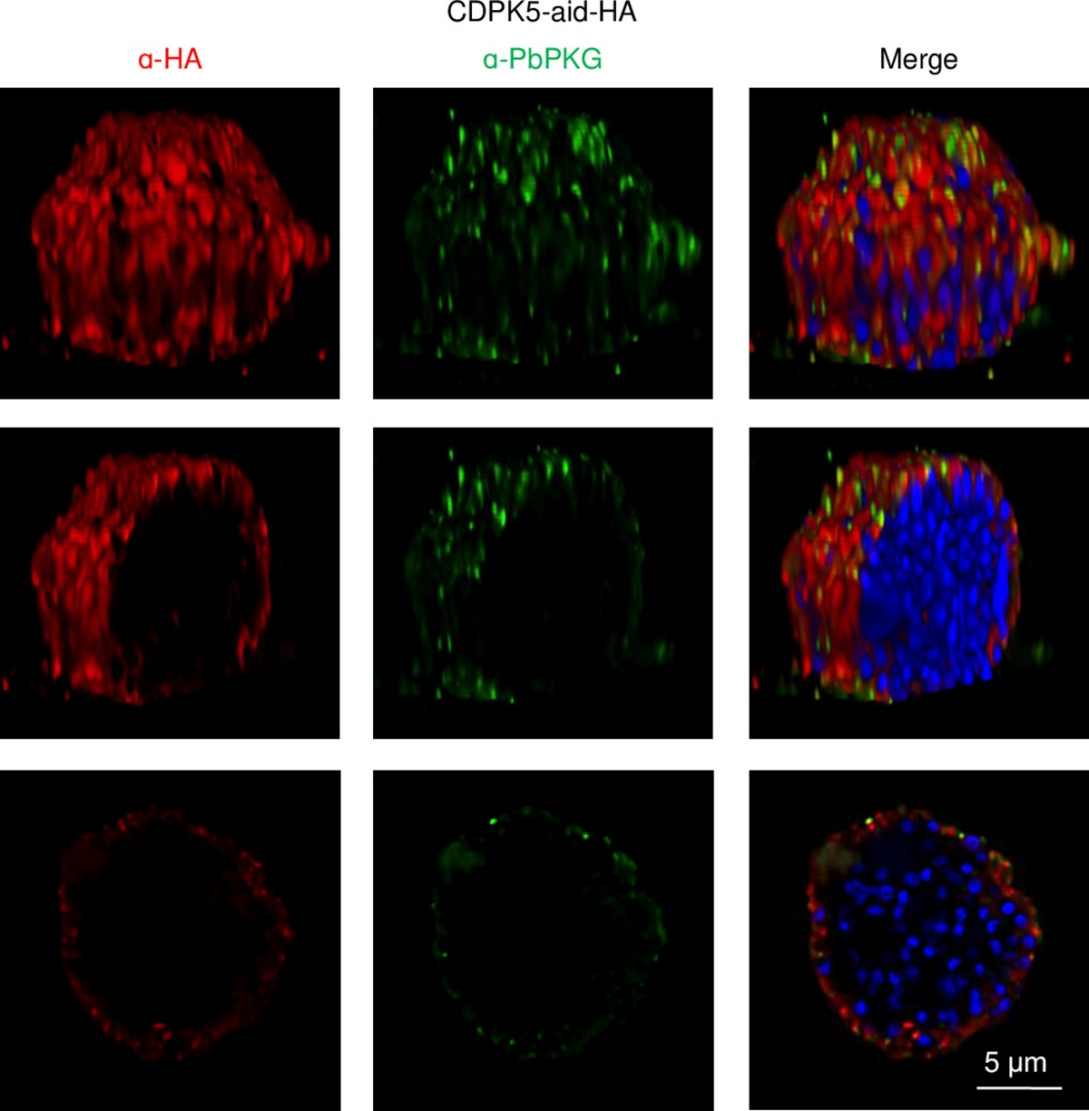
CDPK5 and PKG are present in the periphery of merosomes. Representative deconvolved images and optical sections of immunostained CDPK5-aid-HA merosomes. The top panel illustrates a volumetric view of a merosome. Middle and bottom panels illustrate longitudinal optical sections of the merosome.

**Fig 3:**
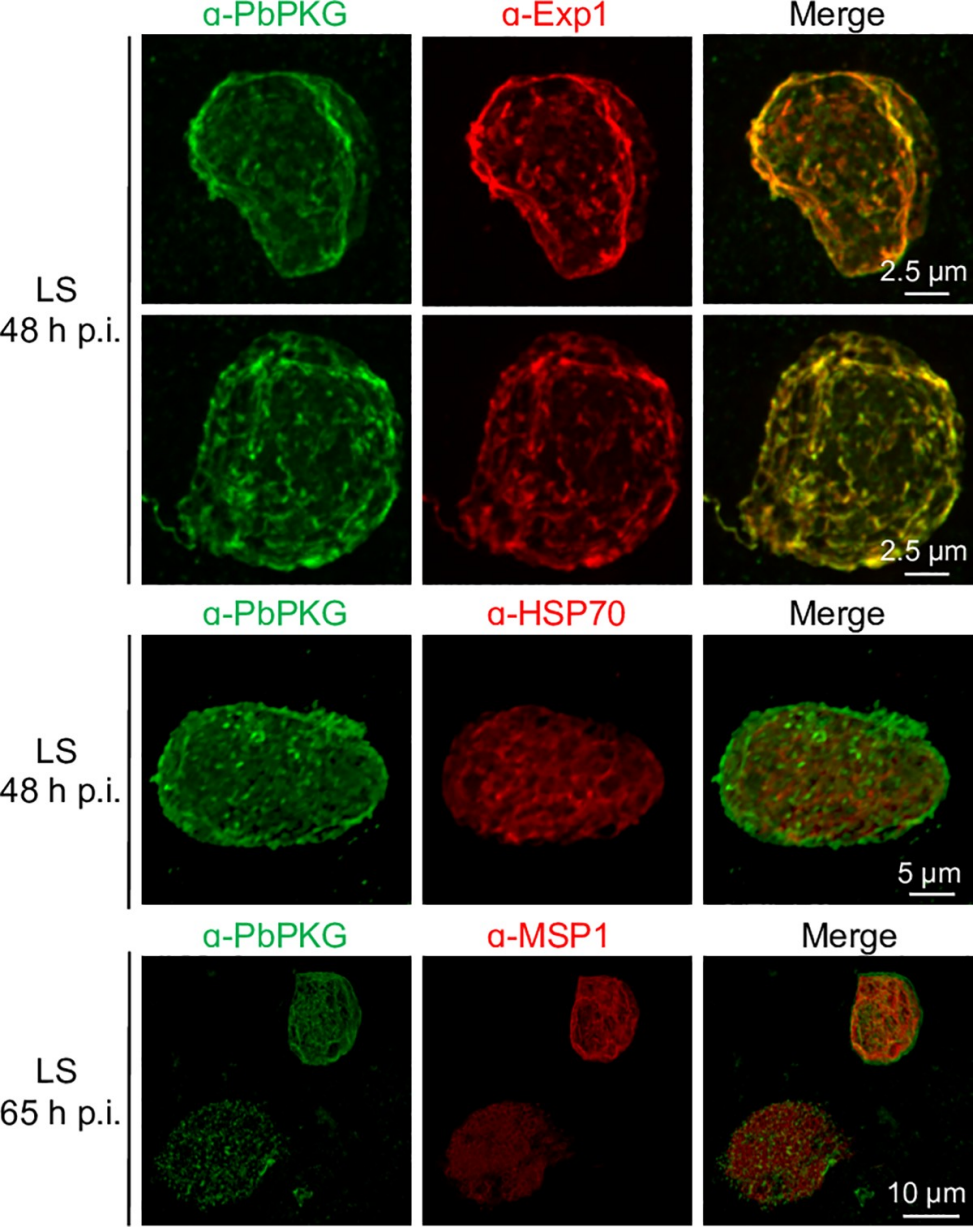
PbPKG localization liver stages. Representative images of PbPKG’s spatial expression in liver stages of *P*. *berghei* parasites expressing luciferase (PbLuc). Exp1 is a resident protein of the parasitophorous vacuole membrane, HSP70 is a cytoplasmic protein and Merozoite Surface Protein (MSP1) is a marker of the parasite plasma membrane.

We began functional interrogation of CDPKs in pre-erythrocytic stages by examining their temporal and spatial protein expression in sporozoites and liver stages. We previously reported the presence of CDPK4 in sporozoites and intracellular liver stage parasites formed 24–65 h post infection (p.i.) of HepG2 cells [19]. Here, we demonstrate that CDPK1 and CDPK5 are also present in sporozoites (Fig 1). CDPK5 is expressed at a low level compared to CDPK1. In liver stages, expression of the three kinases is dynamic. CDPK1 and CDPK5 were not detected in early liver stages 24–48 h p.i (Fig 1). At 65 h p.i., when liver stages are close to exiting the hepatocyte, CDPK5 was detected in a subset of liver stages (Fig 1B, S2A Fig). CDPK5 expression correlated with developmental maturity since, in the same culture, liver stages that express it were significantly larger in size compared to those that did not have detectable CDPK5 (Fig 1C). Appearance of CDPK5 in mature liver stages suggested its expression may coincide temporally with parasite egress from the infected hepatocyte. Therefore, we examined its expression in merosomes and detached cells released at 65–67 h p.i. CDPK5 was detectable in all merosomes/detached cells and appeared to localize to their periphery (Fig 2 middle panel, S2B Fig). PbPKG was also present in merosomes but it did not overlap with CDPK5. Our results confirm proteomic detection of CDPK5 and PKG in *P*. *berghei* merosomes [28]. We have previously shown that PbPKG is required for merosome formation or release [19, 21]. To obtain a better understanding of the subcellular localization of PbPKG in developing liver stages, we carried out co-staining experiments with markers of the parasite cytoplasm (HSP70), parasite plasma membrane (MSP1) and parasitophorous vacuole membrane (Exp1) (Fig 3). PbPKG did not co-localize with HSP70 or MSP1. In some liver stages, PKG appeared to be in close proximity to Exp1 but the significance of these results remains to be determined.

### Validation of AID-mediated conditional protein degradation in pre-erythrocytic stages

**Fig 4:**
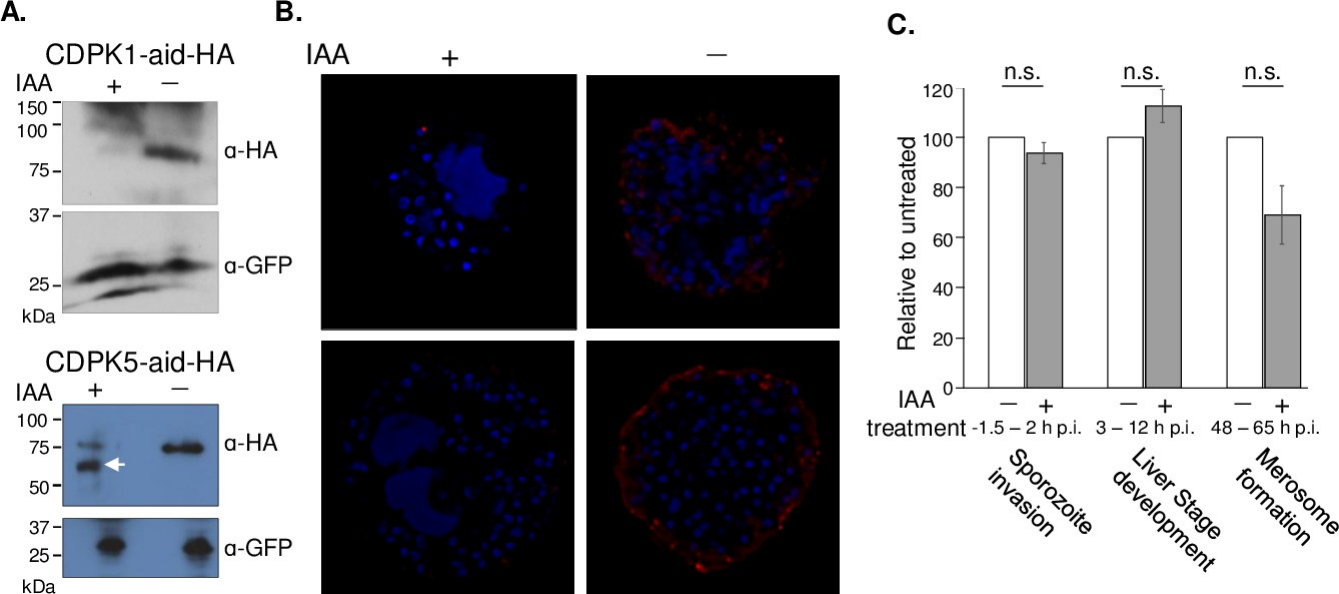
Conditional degradation of AID-tagged proteins is efficient in pre-erythrocytic stages. **A)** Western blot analysis of protein lysates from CDPK1-aid-HA and CDPK5-aid-HA parasites treated with IAA or vehicle. CDPK1-aid-HA and CDPK5-aid-HA were detected using an anti-HA antibody. GFP was used as a loading control. CDPK1-aid-HA and CDPK5-aid-HA have predicted molecular weights of approximately 90 kDa. The arrowhead indicates a byproduct of partial degradation of CDPK5-aid-HA. **B)** Depletion of CDPK5-aid-HA protein in merosomes was examined using immunofluorescence. Optical sections of representative IAA- or vehicle-treated merosomes immunostained with an anti-HA antibody. Parasite nuclei were visualized using DAPI. **C)** Effects of IAA treatment on Ostir1 pre-erythrocytic stages were examined by quantifying sporozoite invasion of HepG2 cells, the number of liver stages detected at 48 h p.i. and the number of merosomes released into the media at 66–70 h p.i., in the presence or absence of IAA. Results shown are average of 2–4 experiments with 3 technical replicates (± SEM), normalized to vehicle-treated samples for each assay. Data were analyzed using an unpaired *t*-test, not significant (n.s.) *P* value > 0.05.

The unique expression profiles of CDPK1, 4 and 5 in pre-erythrocytic stages suggested some overlapping but also distinct roles. We tested the function of the three kinases in pre-erythrocytic stages using conditional protein depletion. We tested the efficiency of IAA-mediated protein degradation in CDPK1-aid sporozoites and CDPK5-aid schizonts. In both cases, Western blot analysis of protein lysates demonstrated a significant loss of AID-tagged proteins (Fig 4A). We confirmed the loss of CDPK5-aid in IAA-treated sporozoites (S3A Fig) and in merosomes/detached cells using immunofluorescence assays (Fig 4B).

Next, we examined if conditions required for protein degradation of AID-tagged proteins have non-specific effects on parasites. We tested the effect of IAA on motility, invasion, infectivity and egress of isogenic control Ostir1 sporozoites, which lack AID-tagged proteins, [27]. Motility was quantified by live imaging sporozoites after treating with IAA or vehicle (S1 Movie and S2 Movie). The percentage of Ostir1 sporozoites that moved in complete circles was similar in both conditions—36% of vehicle-treated (n = 175) and 33% of IAA-treated (n = 165) (S4A Fig). Sporozoite invasion was determined by quantifying the percentage of IAA- and vehicle-treated sporozoites that enter HepG2 cells within 90 min. Their intracellular development was assessed by quantifying the number of liver stages present in HepG2 cells after addition of IAA or vehicle to sporozoite-infected HepG2 cultures 2–14 h p.i. There was no significant effect of IAA treatment on sporozoite invasion or on liver stage development as the fraction of sporozoites that entered cells and the number of liver stages was similar in sporozoites treated with IAA or vehicle (Fig 4C, S4B and S4C Fig). Effect of IAA on parasite egress from hepatocytes was examined by quantifying the number of merosomes present in media of control-infected HepG2 cells treated with IAA 48–65 h p.i. (Fig 4C, S4D Fig). IAA treatment did not significantly affect merosome release by control parasites. We conclude that IAA treatment specifically targets AID-tagged proteins and does not have a deleterious effect on sporozoites. Therefore, IAA-mediated conditional degradation of proteins is a powerful tool for studying protein function in pre-erythrocytic stages of *Plasmodium*.

### CDPK1, CDPK4 and CDPK5 are required for sporozoite motility

*In vitro*, sporozoites display a variety of movement patterns–moving in continuous circles (gliding), waving with one end attached to the substrate, attached with no movement or a combination of the previous three [29]. Motility is accompanied by dynamic changes in intracellular Ca^2+^ [30]. Ca^2+^ flux regulates secretion of adhesins onto the sporozoite surface that mediate attachment to the substrate, and turnover of adhesion sites between the sporozoite and substrate during movement [30, 31]. To examine the role of CDPK1, CDPK4 and CDPK5 as Ca^2+^effectors during motility, we examined the effect of their loss on sporozoite motility.

**Fig 5:**
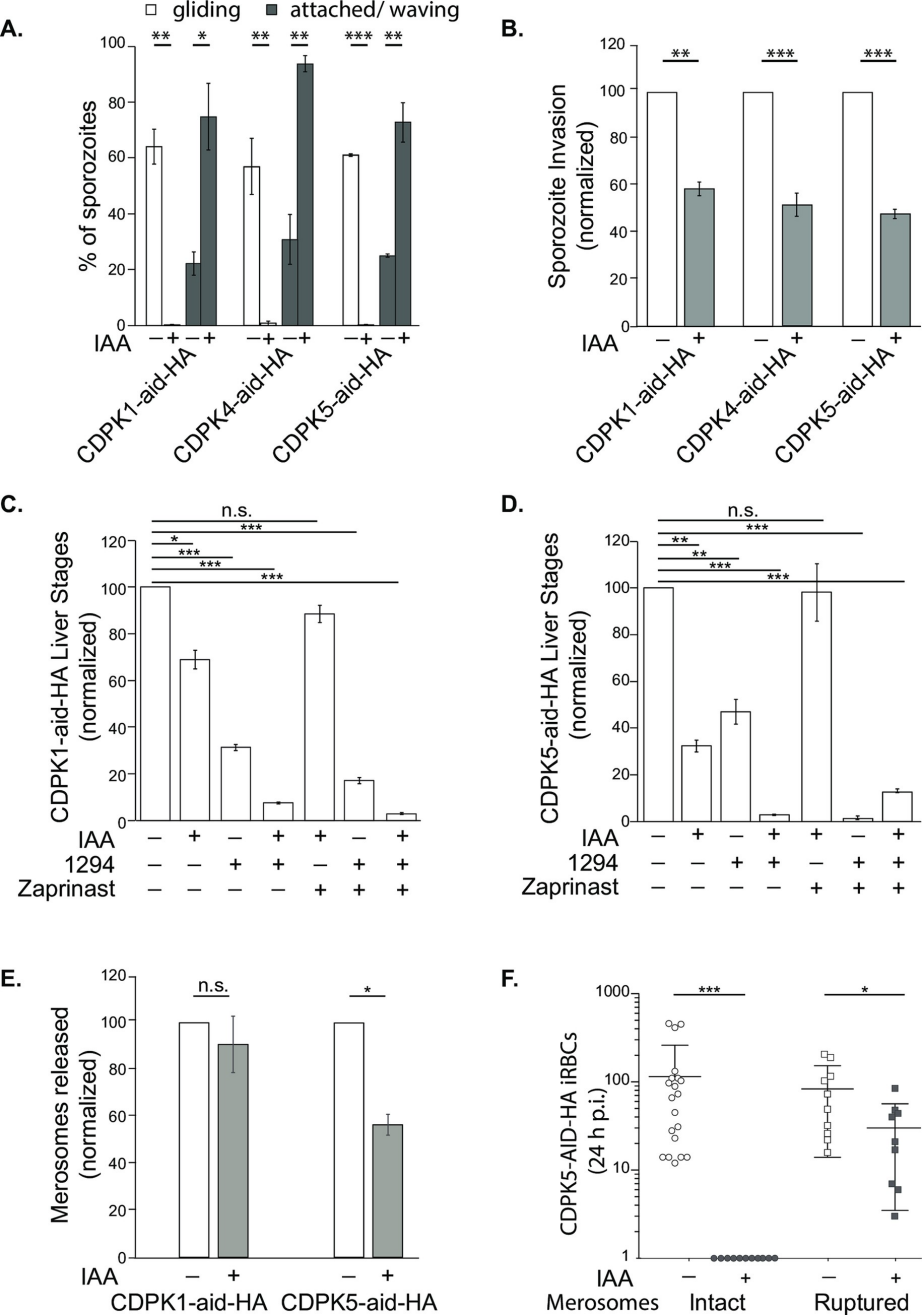
CDPKs have partially overlapping but distinct roles in pre-erythrocytic stages. **A)** Gliding motility of sporozoites was quantified as the percentage of sporozoites that engage in circular motion (± SEM). The experiment was performed 3–4 times for each parasite line. **B)** Sporozoite invasion was examined by determining the percentage of sporozoites that become intracellular within 90 min of addition to HepG2 cells. Results shown are average of 3–4 experiments, each with 3–4 technical replicates (± SEM), normalized to vehicle-treated samples. **C)** Interaction between CDPK1, CDPK4 and PKG in sporozoites was studied by determining the number of liver stages formed by sporozoites in which CDPK1 and CDPK4 functions are inhibited, when PKG is activated. Results shown are average of 3 experiments, each with 3 technical replicates (± SEM), normalized to vehicle-treated samples. **D)** Interaction between CDPK5, CDPK4 and PKG in sporozoites was studied by determining the number of liver stages formed by sporozoites in which CDPK5 and CDPK4 functions are inhibited, in the presence of increased cGMP. Results shown are average of 3 experiments, each with 3 technical replicates (± SEM), normalized to vehicle-treated samples for each assay. **E)** Merosome formation and release was determined by quantifying the number of merosomes present in media of infected cells at 65–67 h p.i. Results shown are average of 2 experiments with CDPK1-aid-HA and 3 experiments with CDPK5-aid-HA, each with 3 technical replicates (± SEM), normalized to vehicle-treated samples. **F)** Infectivity of CDPK5-aid-HA merosomes and hepatic merozoites was quantified by FACS detection of GFP-positive events in 100,000 events at 24 h p.i. (iRBCs ± SEM). The experiment was performed 4 times with intact merosomes (5 mice/each group/experiment), and 2 times with ruptured merosomes (4–5 mice/each group/experiment). Data were analyzed using an unpaired *t*-test or one-way ANOVA, Dunnett’s multiple comparisons test, non-significant (n.s.) *P* > 0.05, * *P* value < 0.05, ** *P* value < 0.005, *** *P* value < 0.0005.

Depletion of CDPK1, CDPK4 or CDPK5 had no significant effect on sporozoite attachment to the substrate but sporozoites lost uninterrupted circular movement (S3 Movie–S8 Movie, Fig 5A, S4E–S4G Fig). Instead, they stayed attached to the substrate with either one pole (‘waving’) or both poles attached or displayed discontinuous motion [29]. These results suggest that the formation and efficient turnover of attachment sites between the sporozoite and the substrate, and consequently sporozoite motility, requires a threshold of Ca^2+^ signaling that is reached through the combinatorial function of each of these kinases. Each kinase can partially but not completely compensate for the loss of another.

*In vivo*, motility enables sporozoites to disseminate from the site of bite in the skin by traversing through cell layers, entering a blood vessel and invading a hepatocyte in the liver. Therefore, we examined if decreased motility that results from CDPK1, 4 or 5 depletion has any functional effect on cell traversal by sporozoites and their eventual invasion of hepatocytes. Depletion of CDPK1 or CDPK5 did not decrease cell traversal by sporozoites (S3B Fig). We were unable to test cell traversal in CDPK4-aid-HA sporozoites due to insufficient sporozoites. Invasion of HepG2 cells decreased by approximately 50% in all IAA-treated sporozoites (Fig 5B, S4H Fig). These data demonstrate that CDPK1, CDPK4 and CDPK5 are required for sporozoite invasion of hepatocytes and they have partially overlapping functions in the process. Optimal invasion by sporozoites requires all three kinases. The ability of sporozoites to invade cells despite depletion for CDPK1 or CDPK4 or CDPK5 and an apparent complete loss of circular motion *in vitro* suggests that motility on glass coverslips is likely an incomplete representation of sporozoite motility on extracellular matrix. The extracellular matrix provides a greater number or variety of adhesins using which sporozoites can achieve sufficient motility. Similar differences in motility on a glass surface [11] or Matrigel were observed in the CDPK3 knockout parasites [10].

Decreased invasion by CDPK1-depleted sporozoites is expected to diminish the number of liver stages formed by these sporozoites. However, previous studies did not find a significant difference in the number of liver stages formed by sporozoites in which the CDPK1 gene was deleted using stage-specific DNA excision (CDPK1 cKO) [8]. To reconcile these seemingly contradictory results, we examined the possibility that CDPK1 protein is not significantly reduced in CDPK1 cKO sporozoites. Using immunofluorescence assays, CDPK1 was detected in these sporozoites (S5 Fig) and its presence was entirely consistent with the normal infectivity of CDPK1 cKO sporozoites. IAA-mediated degradation of CDPK1-aid-HA protein is more efficient than stage-specific DNA excision (Fig 4A) and therefore uncovered CDPK1’s function in sporozoite motility and in host cell invasion.

To determine the effect of simultaneous loss of CDPK1 and CDPK4, we generated a parasite line in which both kinases are tagged with AID-HA (CDPK1-aid-HA/CDPK4-aid-HA). CDPK1-AID-HA/CDPK4-AID-HA parasites were viable in the erythrocytic cycle but they failed to form any salivary gland sporozoites. This negative interaction between modified alleles of CDPK1 and CDPK4 confirms previous reports of epistatic interactions between the kinases during gametogenesis [6].

As an alternative, we utilized a bumped kinase inhibitor compound 1294 [19, 32] to test the effect of simultaneously inhibiting CDPK4, and CDPK1 or CDPK5. Addition of 1294 (2 μM) to IAA-treated CDPK1-aid-HA and CDPK5-aid-HA sporozoites at the time of invasion significantly decreased the number of intracellular liver stages present at 48 h p.i., compared to liver stages formed by sporozoites treated with IAA or 1294 alone (Fig 5C and 5D, S4I and S4J Fig). To confirm 1294’s on-target activity, we utilized parasites carrying a ‘gatekeeper’ mutant of CDPK4, CDPK4 S_147_M [18]. Amino acid substitution at the ‘gatekeeper’ position of CDPK4 prevents access of 1294 to its binding pocket. Infection of HepG2 cells by CDPK4 S_147_M sporozoites was insensitive to treatment by 1294 whereas infection by control sporozoites (gatekeeper mutant of PbPKG, PbPKG T_619_Q) was significantly reduced (S6A Fig). These data demonstrate that the effect of 1294 effect on sporozoites is mediated through inhibition of CDPK4. Further, these results establish that CDPK1, CDPK4 and CDPK5 act together to provide optimal sporozoite invasion.

Since CDPKs act downstream of PbPKG during merozoite invasion of erythrocytes (CDPK4 and CDPK1 [6, 7]) and egress (CDPK5 [4]), we tested if increasing cGMP levels during sporozoite invasion could overcome the loss of CDPK1, CDPK4 or CDPK5. Zaprinast, a phosphodiesterase inhibitor, increases cGMP levels and consequently PbPKG signaling. Its addition reversed the effect of depleting CDPK1 or CDPK5 (Fig 5C and 5D). The number of liver stages formed by CDPK1-depleted or CDPK5-depleted sporozoites in the presence of zaprinast was indistinguishable from vehicle-treated sporozoites. However, zaprinast could not overcome simultaneous inhibition of CDPK4 and either CDPK1 or CDPK5 (Fig 5C and 5D, S4I and S4J Fig). These results suggest that PbPKG acts upstream of both CDPK1 and CDPK5 during sporozoite motility and that CDPK4 functions in a pathway that is, at least in part, independent of PbPKG.

### CDPK5 is required for parasite egress from hepatocytes and from merosomes

Entry of *Plasmodium* into the blood stream after exiting the liver is a two-step process. First, merosomes are extruded from the infected hepatocyte into the blood stream [33, 34]. Second, merosomes are carried intact through the blood stream, before disintegrating, possibly in the lung microvasculature [34], and releasing free hepatic merozoites that initiate the first round of erythrocytic infection. Merosome formation requires parasite PKG [34], the subtilisin, SUB1 [35], the phospholipase, PbPL [36] and the parasite vacuole membrane protein, LISP1 [37] but there is little information about the next step–the release of hepatic merozoites from merosomes.

The presence of CDPK4 and CDPK5 in late liver stages suggests a possible function during parasite egress from hepatocytes. However, a conditional deletion of CDPK4 gene in sporozoites did not demonstrate a clear effect on the number of merosomes released in culture [19]. We attempted to confirm these results using IAA-mediated depletion of CDPK4-aid protein in liver stages but the small number of CDPK4-aid-HA sporozoites made these assays unfeasible. As an alternative, we tested the effect of chemical inhibition of CDPK4 on merosome formation. Addition of 1294 to sporozoite-infected HepG2 cells, at 48–66 h p.i., decreased merosomes released into media at 66 h p.i. (S6B Fig).

Depletion of CDPK5-aid in liver stages, starting 48 h p.i., also reduced the number of merosomes formed at 65h p.i (Fig 5E, S4K Fig). Similar treatment of CDPK1-aid-HA-infected HepG2 cells had no significant effect, which is consistent with lack of detectable CDPK1 in liver stages (Fig 5E). These results demonstrate that Ca^2+^ signaling through CDPK5 is required for parasite exit from hepatocytes. Since CDPK5 is detected in free merosomes (Fig 2), we investigated its function in the release of hepatic merozoites. CDPK5-aid-HA merosomes were treated with either IAA or vehicle prior to being injected intravenously into mice. In order to detect infection by hepatic merozoites, blood parasitemia was monitored at 24 h p.i. This time-period enables the completion of a single asexual cycle in *P*. *berghei*. None of the mice infected with CDPK5-depleted merosomes (n = 20) had detectable parasitemia at 24 h p.i. while all mice infected with vehicle-treated merosomes were positive (Fig 5F). Mice infected with CDPK5-depleted merosomes became patent on day 3 p.i. and the growth rate of asexual parasites was similar in the two groups (S6C Fig). We conclude that loss of CDPK5 in merosomes does not affect replication of asexual stages.

The longer pre-patent period and undetectable parasitemia at 24 h p.i. from CDPK5-depleted merosomes suggested that CDPK5 plays an important role in the release of hepatic merozoites from merosomes and/or in their invasion of erythrocytes. To distinguish between these two possibilities, we tested the effect of CDPK5 depletion of the infectivity of hepatic merozoites. We manually ruptured vehicle- or IAA-treated CDPK5-aid-HA merosomes prior to their injection into mice. In this case, all mice were patent at 24 p.i. and the difference in average parasitemias of the two groups of mice was much smaller (Fig 5F). Together, these results are consistent with, although not proof of, a model in which CDPK5 functions in the rupture of the merosome membrane and release of hepatic merozoites (S7 Fig).

## Discussion

Initiation of the erythrocytic cycle by hepatic merozoites is possibly the least-understood step of the malaria infection cycle. Hepatic merozoites have a single opportunity to infect host cells and their release at the wrong time or place, for example in a non-vascular environment, would prevent or severely debilitate the launch of the erythrocytic cycle. While there is significant understanding at the molecular level of events leading to the release of merozoites from infected erythrocytes, almost nothing is known of how hepatic merozoites are released from merosomes. We provide strong evidence that CDPK5 regulates this process. Breakdown of the merosome membrane is likely a response to environmental cues that result in Ca^2+^ flux in merosomes, activation of CDPK5 and release of proteolytic enzymes.

Depletion of CDPK5 from merosomes does not impair the ability of hepatic merozoites to invade erythrocytes—hepatic merozoites that are manually-released, from CDPK5-depleted merosomes, initiate erythrocytic infection normally. In contrast, intact merosomes depleted for CDPK5 exhibit a significant delay in initiating erythrocytic infection, as blood stage parasitemia was undetectable at 24 h p.i. It has been suggested that arrest of merosomes in the lungs, which have low macrophage density and reduced shear forces in the pulmonary capillary bed from lower blood velocity, facilitates infection of erythrocytes by hepatic merozoites [34]. We hypothesize that interaction between merosomes and a lung-specific receptor could trigger CDPK5-mediated release of hepatic merozoites. Another mechanism that could account for delayed patency of CDPK5-depleted merosomes is better clearance by the host immune system. Testing these models requires development of quantitative assays for release of hepatic merozoites from merosomes. In addition, it will be interesting to explore if the function of CDPK5 during release of hepatic merozoites requires its localization to the merosome periphery.

CDPK5 is also required for the formation of merosomes albeit its depletion has a relatively modest effect on the number of merosomes. One interpretation of these results is that CDPK5 primarily functions after merosomes have been released from the hepatocyte, for example in the release of hepatic merozoites. Alternatively, merosome formation could require overlapping/redundant actions of CDPK5 and CDPK4, since both are present in liver stages at 65 h p.i.. It is also possible that the function of CDPK5 in merosome formation is compensated by PbPKG. We cannot rule out the possibility that IAA-mediated depletion of CDPK5-aid-HA in intracellular and intravacuolar parasite stages, such as liver stages, is less efficient compared to its depletion in sporozoites or released merosomes. Incomplete depletion of CDPK5 in liver stages could provide sufficient protein for close-to-normal function in merosome formation.

The relative dearth of knowledge about the function of CDPKs in pre-erythrocytic stages can be attributed, in part, to the technical challenge of functionally analyzing kinases that are indispensable in asexual and sexual stages. Despite this difficulty, it is important to understand functions of CDPKs in these stages because they constitute the first step in mammalian infection, and the study of CDPKs in these stages could identify targets for drugs that prevent or treat *Plasmodium’s* liver infection. In addition, these studies will provide a fuller view of the stage-specific and stage-transcending functions of different family members. The relative contribution of different CDPK family members in different parasite stages may be determined by the threshold of Ca^2+^ signaling required for different cellular processes, the sensitivity of specific kinases to Ca^2+^ level, their expression levels and their subcellular localization.

Our work reveals nuances of CDPK signaling in different parasite stages. First, our results demonstrate a new stage-specific role for CDPK5—in sporozoite motility and consequently parasite invasion of hepatocytes. CDPK5 has not been implicated in RBC invasion by merozoites [4]. In contrast to its role in parasite invasion, CDPK5’s role in parasite egress is stage-transcending as it is required for exit from both erythrocytes and hepatocytes. During the erythrocytic cycle, CDPK5 triggers micronemal secretion leading to merozoite egress from schizonts [4]. The mechanism whereby CDPK5 enables release of hepatic merozoites from merosomes remains to be investigated. In addition, its localization in late liver stages and merosomes needs to be fully validated. Super-resolution microscopy, that is currently unavailable to us, could distinguish between the cytoplasm, parasite plasma membrane, vacuole membrane and merosome membrane. Subcellular localization of CDPKs and PKG may be dynamic in sporozoites and liver stages. A careful investigation their spatiotemporal patterns will deepen our understanding of their functions in pre-erythrocytic stages. If future work confirms localization of CDPK5 in merosomes, it is tempting to speculate that the kinase participates in a spatially-restricted protease cascade close to the merosome surface, thereby facilitating the release of hepatic merozoites.

Second, our results demonstrate that while different invasive stages may require the same set of CDPK kinases, the relative contribution of specific kinase members can vary at different stages or during specific processes. These differences may reflect the diverse host-environments encountered by different stages. We show that CDPK1, CDPK4 and CDPK5 have distinct roles in sporozoite motility and that these functions are only partially overlapping. Depletion of any of these kinases significantly reduces sporozoite motility. Since IAA-treated sporozoites attach to the surface, we speculate that their defective motility is likely due to dysregulation in turnover of adhesion sites that is required for sporozoites to move forward [31]. In contrast, in ookinetes, individual depletion or inhibition of CDPK1 and CDPK4 does not affect motility *in vitro* and only simultaneous loss significantly reduces ookinete speed [6]. The implication is that each kinase can fully compensate for the other during ookinete traversal [6]. As shown previously, ookinete motility and traversal through the midgut epithelium relies most heavily on CDPK3 [11].

The different effects of genetic knockdown and chemical inhibition of CDPK4 suggest that parasites can adapt to genetic ablation of CDPK4 in sporozoites but cannot adapt to its chemical inhibition. In yeast and mammalian cells, there are several examples of kinase inhibitors and genetic mutants producing different functional outcomes [38]. Compensatory pathways in genetic mutants can arise from increased activity of a related kinase, acquisition of suppressor mutations or disruption of a protein complex [38]. Since chemical inhibition of kinase activity is rapid, it may not allow sufficient time for compensatory pathways that can develop during differentiation from sporozoites into merosomes.

We found that, despite loss of continuous movement, sporozoites depleted of CDPK1 or CDPK5 maintained the ability to traverse through cells but were significantly attenuated in invasion. Our observations add to the growing body of work demonstrating the lack of absolute correlation between motility *in vitro* and cell invasion by sporozoites. *P*. *berghei* mutants in profilin display profound motility defects but invade and infect hepatocytes normally *in vivo* [39]. Similarly, despite a total loss of circular movement *in vitro*, sporozoites lacking TRAP are not abrogated in invasion of salivary glands or of hepatocytes [40]. On the other hand, *P*. *berghei* sporozoites carrying mutations in the N-terminal domain of actin display normal gliding motility *in vitro* but have a significant defect in salivary gland invasion [41]. Interestingly, these mutant sporozoites infect mice normally demonstrating that invasion of salivary glands is not identical to the invasion of mammalian cells [41]. These data indicate that motility, cell traversal and invasion are complex processes that do not rely on all the same molecules. Sporozoites traverse through cells by forming a transient vacuole without the formation of a tight junction between the sporozoite and hepatocyte membranes [42]. Productive invasion requires the formation of a tight junction and sporozoites are contained within a parasitophorous vacuole. Since loss of CDPK1 and CDPK5 reduces only invasion, it implies that residual motility in depleted sporozoites is sufficient for traversal. One explanation is that CDPK1 and CDPK5 could regulate the formation of the tight junction or another process specific to the form of cell entry that is accompanied by the formation of a permanent vacuole.

Optimal invasion by sporozoites requires a network of CDPKs–CDPK1, CDPK4 and CDPK5, as shown here, and CDPK6 as shown previously [12]. We posit that, *in vivo*, reduced motility that results from inhibition or loss of CDPK1, CDPK4 or CDPK5 will severely impair cell entry by sporozoites, thereby attenuating hepatocyte infection. In contrast, during invasion by asexual stages, the loss of CDPK1 or CDPK4, individually or together, has no significant effect. The role of CDPK4 in erythrocytic invasion is seen only in the background of reduced PKG activity [6]. The role of CDPK1 in invasion is uncovered only in the background of simultaneous reduction in PKG and CDPK4 activities. It is possible that erythrocytic stages adapt to the loss of CDPK1 and CDPK4 by upregulating the activity of another CDPK member or of PKG.

We have also determined that CDPK4 and CDPK5 play a role in merosome formation (Fig 5E and S6B Fig). Although conditional deletion of the CDPK4 gene in sporozoites (CDPK4 cKO) did not significantly decrease merosome formation [19], its chemical inhibition has a modest effect. The lack of merosome reduction in CDPK4 cKO parasites may be attributed to upregulation of compensatory pathways. To test these models, we wanted to determine the effect on merosome formation of rapid reduction in CDPK4 protein. We were unable to do so because CDPK4-aid-HA sporozoites are not produced in sufficient numbers for robust merosome assays. Further studies using alternative methods for depleting CDPK4 exclusively in liver stages and compounds with greater specificity for CDPK4 will address this issue.

## Materials and methods

### Ethics statement

All animal work in this project was reviewed and approved by the Institutional Animal Care and Use Committee (IACUC) of Rutgers New Jersey Medical School, approval number TR201900067, following guidelines of the Animal Welfare Act, The Institute of Laboratory Animal Resources Guide for the Care and Use of Laboratory Animals, and Public Health Service Policy.

### Construction of AID-HA tagged parasite lines

The targeting plasmid for modifying CDPK4 with the aid-HA degron was constructed by amplifying a C-terminal fragment of CDPK4 using PCR primers AATTGGAGCTCCAC**CGCGG**CAAGTATTAAGTGGTATTACATATATG and TCATTCTAGT**CTCGAG**ATAGTTACATAGTTTTATTAACATGTCTC. The PCR product was cloned into the previously described AID-tagging plasmid expressing mCherry (pG364) [27], using SacII and XhoI. The targeting plasmid for modifying CDPK5 with the aid-HA degron was constructed by cloning a fragment amplified using PCR primers AATTGGAGCT-CCA**CCGCGG**CATAGAGATTTAAAGCCAGAA and TCATTCTAGT**CTCGAG**AGATTGT-CTTCCAGACATC, into a previously described AID-tagging plasmid expressing GFP (pG362) [27]. The CDPK4-aid-HA targeting plasmid was linearized using BspEI and the CDPK5-aid-HA targeting plasmid was linearized using BclI. Linearized plasmids were transfected into the previously described OsTIR1-expressing parent line [27], using standard procedures [43]. Transfected parasites were selected by pyrimethamine treatment and clonal lines were established through limited dilution. Modification of CDPK4 was confirmed using PCR primer pairs P1 (TGAAGTAGATGCAGCTAG) + P2 (GTTAAATGTGGGGTAAAAAA) and P3 (GTATTTACCCTGTCATACAT) + P4 (GATTAAGTTGGGTAACGC). Modification of CDPK5 was confirmed using PCR primer pairs P2 + P5 (CAAATGGATCATCCAAATATT) and (P3) + P6 (AAGGAATAGAAGGTAGAAATTG).

### Mosquito infections

*Anopheles stephensi* mosquitoes infected with PbLuc [44] were obtained from the insectary at the New York University School of Medicine. Uninfected *A*. *stephensi* were obtained from the New York University School of Medicine and the Johns Hopkins University School of Public Health. They were fed on infected Swiss-Webster mice. Mosquitoes were maintained on 20% sucrose at 25°C at a relative humidity of 75–80%. Sporozoites were obtained by crushing salivary glands dissected on days 18–25 post-feeding. Sporozoites were counted in a hemocytometer.

### Immunofluorescence assays for protein detection in sporozoites, liver stages and merosomes

Primary antibodies used were: anti-HA (Biolegend, 1:200–1:400), anti-PKG (1:1000 [19]), anti-PfCDPK1 (# 2129, 1:1000, kind gift of Dr. Barbara Kappes [45]) anti-CS (3D11, 1 μg/mL), anti-HSP70 (2E4, 1.0 μg/mL [46]), anti-Exp1 (1:1000, kind gift of Dr. Volker Heussler) and anti-MSP1 (1:400, kind gift of Dr. Anthony Holder). Secondary antibodies (anti-mouse Alexa488, anti- rabbit Alexa594 and anti-chicken Alexa 594, Molecular Probes) were used at a dilution of 1:3000.

Sporozoites (0.5–1 x 10^6^) were purified as previously described [47], air-dried at room temperature on poly L-lysine coated glass slides. They were fixed in 4% PFA for 20 min at RT, permeabilized with 0.5% TritonX-100 for 15min at RT before blocking with 3% BSA in PBS for 1h. Primary antibodies diluted in blocking solution were added at the appropriate dilutions and incubated either for 1h at RT or overnight for 4°C. Secondary antibodies diluted in blocking solution were incubated for 1h at RT. Washes with PBS were performed after incubation with each antibody.

Protein expression in liver stages was examined by infecting HepG2 cells, at 70% confluency, with sporozoites (2x10^4^–4 x10^4^). At the appropriate time post-infection, cells were fixed in 4% PFA for 20 min, permeabilized with cold methanol for 15min and blocked in 3% BSA/PBS for 1 h. For MSP1 staining, cells were permeablized with 0.1% TritonX-100. Primary antibodies diluted in blocking solution were added at the appropriate dilutions and incubated either for 1 h at RT or overnight for 4°C. Cells were washed 3 times with PBS. Secondary antibodies diluted in blocking solution were added at the appropriate dilutions and incubated for 1 h at RT.

Protein expression in merosomes was examined by infecting HepG2 cells, at 70% confluency, with sporozoites (8x10^4^–10 x10^4^). Medium was replaced every 12h. Detached cell-containing cell culture supernatant was collected at 65–67 h p.i. and treated with either IAA (500 μM) or vehicle (1% ethanol). After 90 min at RT, cells were allowed to settle onto polyL-Lysine coated slides, fixed in 4% PFA for 20 min and permeabilized with cold methanol for 15 min before treatment with 3% BSA/PBS for 1 h for blocking. Antibodies were added as described above.

Images were captured using a Nikon A1R laser scanning confocal microscope using 60X/NA1.4 oil objective. Image deconvolution was performed using the Nikon NIS Elements Advanced Research software. For measuring the diameter of liver stages, images were taken at random using a 100x objective on an Olympus BX61 microscope. The area of a region-of-interest was measured using Olympus CellSens software.

### Western blot analysis for protein detection

Sporozoites (0.5x10^6^ CDPK1-aid-HA/treatment) and schizonts (2.5x10^6^ CDPK5-aid-HA/treatment) were lyzed in Laemlii buffer in the presence of protease inhibitor. Protein lysates were separated on a 12% SDS-PAGE gel and transferred to a PVDF membrane using standard wet transfer. The membrane was blocked in 3% BSA in PBS containing 0.1% Tween20. Anti-HA (Biolegend, 1:1000) and anti-GFP (Rockland Laboratories, 1:1000) antibodies were incubated overnight at 4°C. HRP-conjugated secondary antibodies (GE HealthSciences) were incubated for 1 h at RT. The membrane was washed (3 x15 min) after primary and secondary antibodies in PBS containing 0.1% Tween20. Membrane was developed using the SuperSignal substrate (Thermofisher Scientific).

### Motility assays

Sporozoites dissected in DMEM (1x10^4^) were incubated at RT for 90 min with either IAA (500 μM) or vehicle (1% ethanol), in a volume of 25 μL. After addition of equal volume of 6% BSA, they were transferred to a 96-well plate with an optical bottom and centrifuged for 3 min at 4°C. Sporozoites were filmed on a Nikon A1R laserscanning confocal microscope using a 20X/NA0.75 objective at 37°C. Movies were recorded over 90 frames at 1 Hz. Image acquisition and analysis was performed using NIS Elements software from Nikon. Fluorescence intensity projections were processed using NIS Elements and movement patterns were determined through visual inspection of individual sporozoites.

### Cell traversal assays

Cell traversal assays were performed as previously described [48]. Briefly, sporozoites (4 x10^4^/well), in plain DMEM medium, were treated with either IAA (500 μM) or vehicle (1% ethanol) in a volume of 150 **μ**L. After 90 min at RT, sporozoites were added to confluent HepG2 cells plated in 8-chamber LabTek slides in the presence of fluorescein-conjugated dextran (1 mg/mL). After incubation at 37°C for 1 h, cells were washed in PBS and fixed with 4% PFA.

### Sporozoite invasion assays

Sporozoites (4 x10^4^/well), in plain DMEM medium, were treated with either IAA (500 μM) or vehicle (1% ethanol) in a volume of 75 μL. After incubation for 90 min at RT, 75 μL of 2% BSA in DMEM was added along with either IAA (500 μM) or vehicle (1% ethanol). Sporozoites (150 μL/well) were added to confluent HepG2 cells plated in 8-chamber LabTek slides. Invasion assays were performed as previously described [48]. Briefly, 90 min after sporozoite addition, cells were fixed with 4% PFA, blocked with 3% BSA in PBS and incubated with 3D11 (1μg/mL) for 1h at RT. After washes with PBS, cells were incubated with anti-mouse Alexa594 (1:3000) for 1h at RT. Cells were permeablized with cold methanol for 15 min, blocked and incubated with 3D11. After washes, cells were incubated with anti-mouse Alexa488 (1:3000) for 1h at RT. The number of sporozoites that became intracellular were determined by calculating the difference in numbers of sporozoites that stain with Alexa488 and Alexa594. The percentage of sporozoites that invaded was calculated by determining the percentage of total sporozoites that were intracellular.

### Sporozoite infection assays

Sporozoites, pre-treated with IAA (500 μM), vehicle (1% ethanol), 1294 (2 μM) and zaprinast (50 μM), were added to HepG2 cells, for a final volume of 200 μL. Sporozoite-containing medium was replaced with complete DMEM at 2 h p.i. and 24 h p.i. Cells were fixed with 4% PFA at 48 h p.i., permeablized with cold methanol for 15 min, blocked for 1 h with 3% BSA in PBS before incubation with the anti-HSP70 and anti-mouse Alexa488 antibodies as described above. The number of liver stages was determined through microscopic examination.

### Liver stage development

Sporozoites (2–4 x10^4^/well), in DMEM supplemented with 10% FCS, were added to HepG2 cells plated in 8-chamber LabTek slides. To determine its effect on liver stage development, IAA or vehicle was added to infected HepG2 cells 3 h p.i.. Medium was replaced at 14 h p.i. and cells fixed for immunofluorescence analysis using anti-HSP70 as described above.

### Merosome formation

Sporozoites (8–10 x10^4^/well) were added to sub-confluent HepG2 cells plated on glass coverslips in 24-well plates. Medium was replaced every 12 h. To determine its effect on the development and release of merosomes, IAA was added to infected HepG2 cells 48 h p.i.. The number of merosomes present in the medium was quantified in a hemocytometer at 65–67 h p.i..

### *In vivo* infections

Merosomes were treated with IAA (500 μM) or vehicle (1% ethanol) for 90 min at RT in DMEM. Following treatment, they were injected intravenously into Swiss-Webster female mice (4–5 mice/group, 6–8 weeks) either immediately (200 intact merosomes/mouse) or after passaging 10 times through a 23G needle (5000 ruptured merosomes/mouse) as previously described [49]. Blood was collected from mice 24 h p.i. and analyzed by flow cytometry for the number of GFP-positive events as previously described [50]. A total of 1x10^5^ events was counted per sample. Parasitemia was confirmed through daily microscopic examination of Giemsa-stained blood smears.

### Statistical analysis

Data were examined using GraphPad Prism v7.

## Supporting information

S1 Movie Video of vehicle-treated Ostir1 sporozoites. (MP4)

S2 Movie Video of IAA-treated Ostir1 sporozoites. (MP4)

S3 Movie Video of vehicle-treated CFP-expressing CDPK1-aid-HA sporozoites. (MP4)

S4 Movie Video of IAA-treated CFP-expressing CDPK1-aid-HA sporozoites. (MP4)

S5 Movie Video of vehicle-treated mCherry-expressing CDPK4-aid-HA sporozoites. (MP4)

S6 Movie Video of IAA-treated mCherry-expressing CDPK4-aid-HA sporozoites. (MP4)

S7 Movie Video of vehicle-treated GFP-expressing CDPK5-aid-HA sporozoites. (MP4)

S8 Movie Video of IAA-treated GFP-expressing CDPK5-aid-HA sporozoites. (MP4)

S1 Fig Characterization of CDPK4-aid-HA and CDPK5-aid-HA parasites. **A)** Schematic for modifying CDPK4 and CDPK5 with aid-HA_2x_ through single recombination. The blue box represents the expression cassette for hDHFR and fluorescent marker and the dashed line represents the site of plasmid linearization. Integration of targeting constructs was detected by interrogating the locus by PCR. Primer pairs P1+P2 and P3+P4 were used to detect 5’ and 3’ integration events in CDPK4-aid-HA parasites, respectively. Primer pairs P5+P2 and P3+P6 were used to detect 5’ and 3’ integration events in CDPK5-aid-HA parasites, respectively. **B)** Southern hybridization strategy for detecting CDPK4-aid-HA and CDPK5-aid-HA modification. Restriction-digested parasite genomic DNA from CDPK4-aid-HA (SphI + XhoI) or CDPK5-aid-HA (XbaI) was probed with DIG-labeled probes (indicated by grey boxes). Digested WT genomic DNA was used as control. **C)** Sporozoite numbers in salivary glands of mosquitoes infected with CDPK1-aid-HA, CDPK4-aid-HA, CDPK5-aid-HA and isogenic Ostir1 parasites used as control. The experiment was repeated 3–5 times.
(TIF)

S2 Fig CDPK5 localization in pre-erythrocytic stages of CDPK5-aid-HA parasites. CDPK5 was detected using an anti-HA antibody. Anti-PbPKG was used as control. **A)** CDPK5 localization in mature liver stages at 65 h p.i. **B)** Representative deconvolved images and optical sections of immunostained CDPK5-aid-HA merosomes. The top panel illustrates a volumetric view of a merosome. Middle and bottom panels illustrate longitudinal optical sections of the merosome.
(TIF)

S3 Fig **A)** Representative images of CDPK5 expression in CDPK5-aid-HA sporozoites after IAA treatment. IAA depletes CDPK5 protein in sporozoites. **B)** Cell traversal by sporozoites is not significantly affected by depletion of CDPK1 or CDPK5. The number of cells containing dextran-FITC (± SEM) formed in each condition was normalized to vehicle-treated controls. The experiment was performed 3–4 times with 4 technical replicates. Results shown are average of 3–4 experiments with 3 technical replicates (± SEM), normalized to vehicle-treated samples.
(TIF)

S4 Fig IAA-dependent depletion of CDPKs on pre-erythrocytic stages. A-D) Effect of IAA treatment on gliding (A), invasion (B), liver-stage formation (C) and merosome formation (D) by Ostir1 control parasites. Results shown are the mean of 2–4 experiments with 3 technical replicates (± SD). E-G) Effect of IAA-treatment on gliding by CDPK1-aid-HA (E), CDPK4-aid-HA (F) and CDPK5-aid-HA (G) sporozoites. Results shown are the mean of 3–4 experiments (± SD). H) Effect of IAA-treatment on invasion by CDPK1-aid-HA, CDPK4-aid-HA and CDPK5-aid-HA sporozoites. Results shown are the mean of 3–4 experiments, each with 3–4 technical replicates (± SD). I) Effect of simultaneous depletion of CDPK1-aid-HA protein and inhibition of CDPK4 activity or enhancement of PKG activity. Results shown are the mean of 3 experiments, each with 3 technical replicates (± SD). J) Effect of simultaneous depletion of CDPK5-aid-HA protein and inhibition of CDPK4 activity or enhancement of PKG activity. Results shown are the mean of 3 experiments, each with 3 technical replicates (± SD). K) Effect of IAA-treatment on merosome formation by CDPK1-aid-HA and CDPK5-aid-HA liver stages. Results shown are the mean of 2 experiments with CDPK1-aid-HA and 3 experiments with CDPK5-aid-HA, each with 3 technical replicates (± SD). Data were analyzed using an unpaired *t*-test or one-way ANOVA, Dunnett’s multiple comparisons test, non-significant (n.s.) *P* > 0.05, * *P* value < 0.05, ** *P* value < 0.005, *** *P* value < 0.0005.
(TIF)

S5 Fig Detection of CDPK1 protein in CDPK1 cKO sporozoites. CDPK1 cKO sporozoites were generated through FlpL-mediated deletion of the CDPK1 ORF in FlpL-expressing parasites (FlpL/TRAP). PbCDPK1 was detected using an anti-PfCDPK1 antibody. Anti-CS was used as control. Images shown are representative of CDPK1 expression in these sporozoites.
(TIF)

S6 Fig Effect of CDPK4 inhibition and CDPK5 depletion on parasite exit from hepatocytes. **A)** Specificity of 1294 in sporozoites was interrogated by testing its effects on HepG2 infection by ‘gatekeeper’ mutants of PKG (T_619_Q) and CDPK4 (S_147_M). Liver stages were quantified at 48 h p.i. The number of liver stages formed under each condition was normalized to vehicle-treated controls. Results shown for CDPK4 S_147_M are average (± SEM) of 3 experiments, each with 3–4 technical replicates. Results shown for PbPKG T_619_Q are average (± SD) of one experiment with 4 technical replicates. **B)** Dose-dependent inhibition of merosome formation by 1294. Merosomes and detached cells were quantified at 65–68 h p.i. with PbLuc sporozoites. Compound was added to infected HepG2 cultures at 48 h p.i. and refreshed every 12 h. The number of merosomes/detached cells (± SEM) formed in each condition was normalized to vehicle-treated controls. The experiment was performed twice with technical triplicates. **C)** Growth rate of CDPK5-aid-HA parasites in erythrocytes. Parasitemias (± SD) of mice infected with vehicle- or IAA-treated CDPK5-aid-HA merosomes were determined daily. Data are from a representative experiment (5 mice/group). Data were analyzed using an unpaired *t*-test, non-significant (n.s.) *P* > 0.05, * *P* value < 0.05, ** *P* value < 0.005, *** *P* value < 0.0005.
(TIF)

S7 Fig Model of CDPK functions in pre-erythrocytic stages. CDPK family members play a critical role during several steps of hepatocyte infection. Sporozoite motility and consequently infection of hepatocytes requires CDPK1, 4 and 5. In addition, CDPK4 and 5 function in the formation or release of merosomes from the infected hepatocyte. The release of hepatic merozoites from merosomes requires CDPK5.
(TIF)
