# Supplementary figures and images for: Overlapping and distinct roles of CDPK family members in the pre-erythrocytic stages of the rodent malaria parasite, Plasmodium berghei
Source: PLoS Pathog. 2020 Aug 31;16(8):e1008131. doi: 10.1371/journal.ppat.1008131 (PMC7485973; doi:10.1371/journal.ppat.1008131)

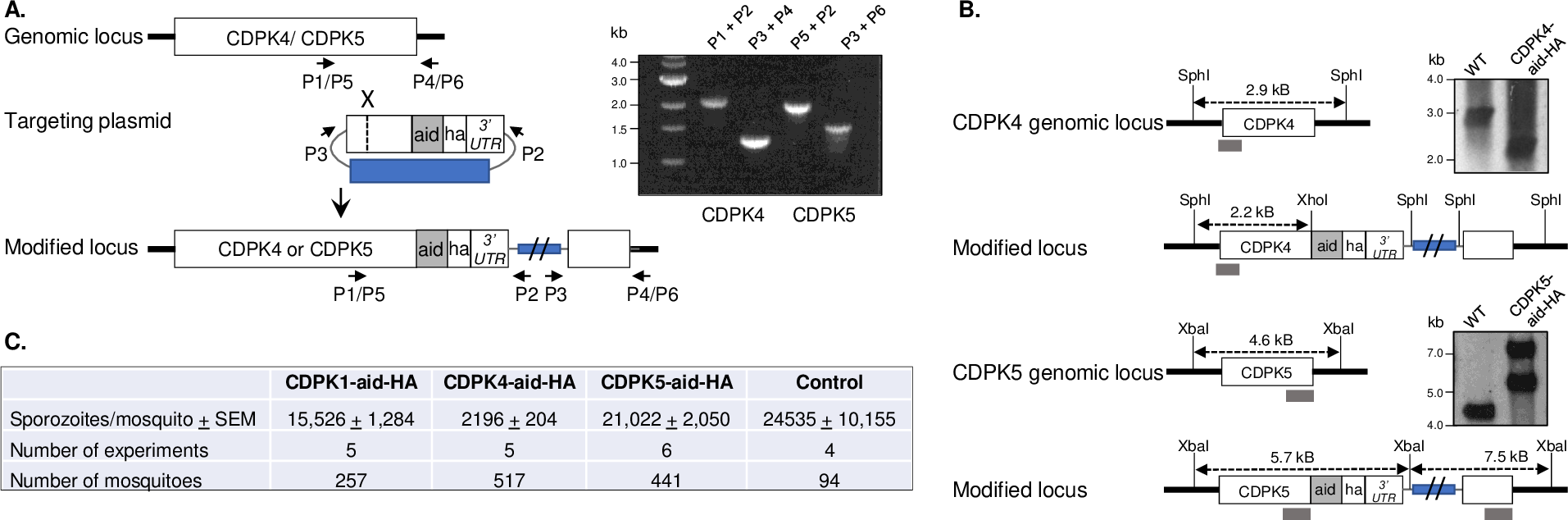

Supplement: S1 Fig — A) Schematic for modifying CDPK4 and CDPK5 with aid-HA2x through single recombination. The blue box represents the expression cassette for hDHFR and fluorescent marker and the dashed line represents the site of plasmid linearization. Integration of targeting constructs was detected by interrogating the locus by PCR. Primer pairs P1+P2 and P3+P4 were used to detect 5’ and 3’ integration events in CDPK4-aid-HA parasites, respectively. Primer pairs P5+P2 and P3+P6 were used to detect 5’ and 3’ integration events in CDPK5-aid-HA parasites, respectively. B) Southern hybridization strategy for detecting CDPK4-aid-HA and CDPK5-aid-HA modification. Restriction-digested parasite genomic DNA from CDPK4-aid-HA (SphI + XhoI) or CDPK5-aid-HA (XbaI) was probed with DIG-labeled probes (indicated by grey boxes). Digested WT genomic DNA was used as control. C) Sporozoite numbers in salivary glands of mosquitoes infected with CDPK1-aid-HA, CDPK4-aid-HA, CDPK5-aid-HA and isogenic Ostir1 parasites used as control. The experiment was repeated 3–5 times. (TIF) [file ppat.1008131.s009.tif]

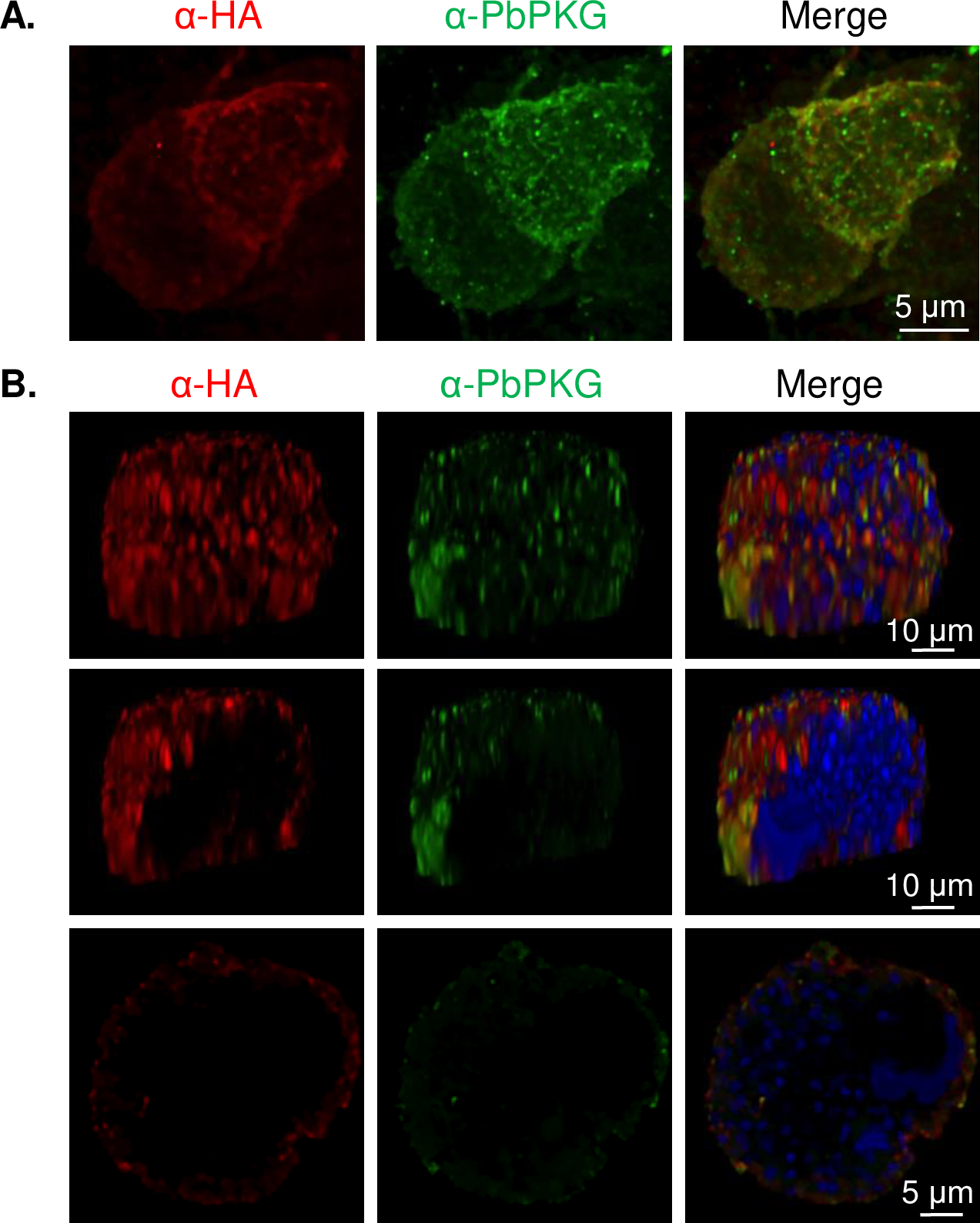

Supplement: S2 Fig — A) CDPK5 localization in mature liver stages at 65 h p.i. B) Representative deconvolved images and optical sections of immunostained CDPK5-aid-HA merosomes. The top panel illustrates a volumetric view of a merosome. Middle and bottom panels illustrate longitudinal optical sections of the merosome. (TIF) [file ppat.1008131.s010.tif]

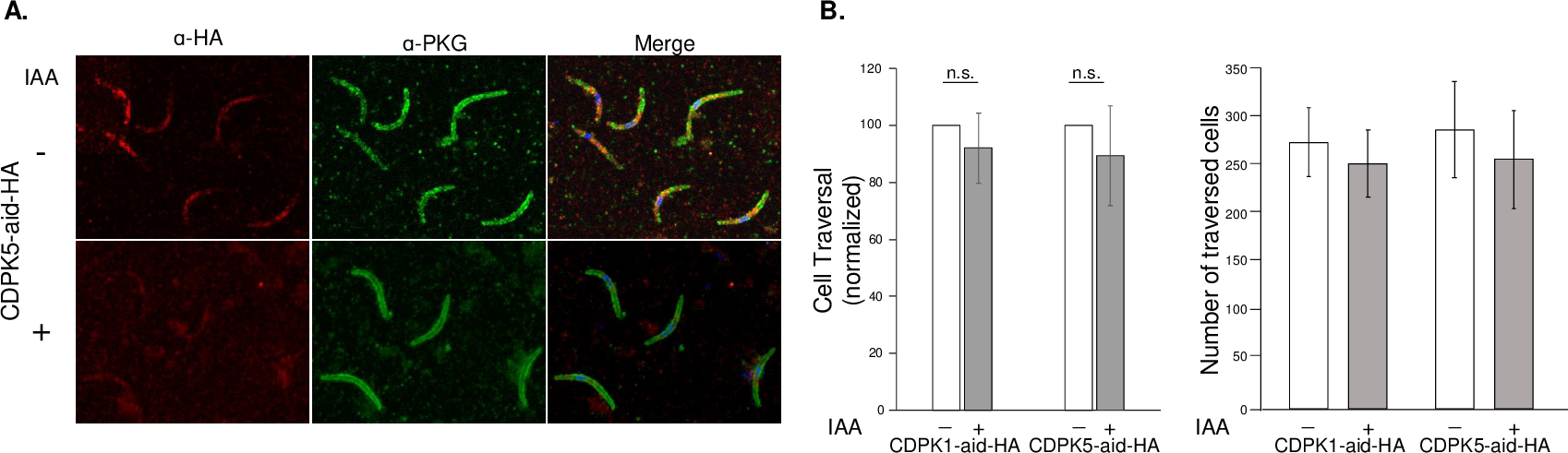

Supplement: S3 Fig — A) Representative images of CDPK5 expression in CDPK5-aid-HA sporozoites after IAA treatment. IAA depletes CDPK5 protein in sporozoites. B) Cell traversal by sporozoites is not significantly affected by depletion of CDPK1 or CDPK5. The number of cells containing dextran-FITC (± SEM) formed in each condition was normalized to vehicle-treated controls. The experiment was performed 3–4 times with 4 technical replicates. Results shown are average of 3–4 experiments with 3 technical replicates (± SEM), normalized to vehicle-treated samples. (TIF) [file ppat.1008131.s011.tif]

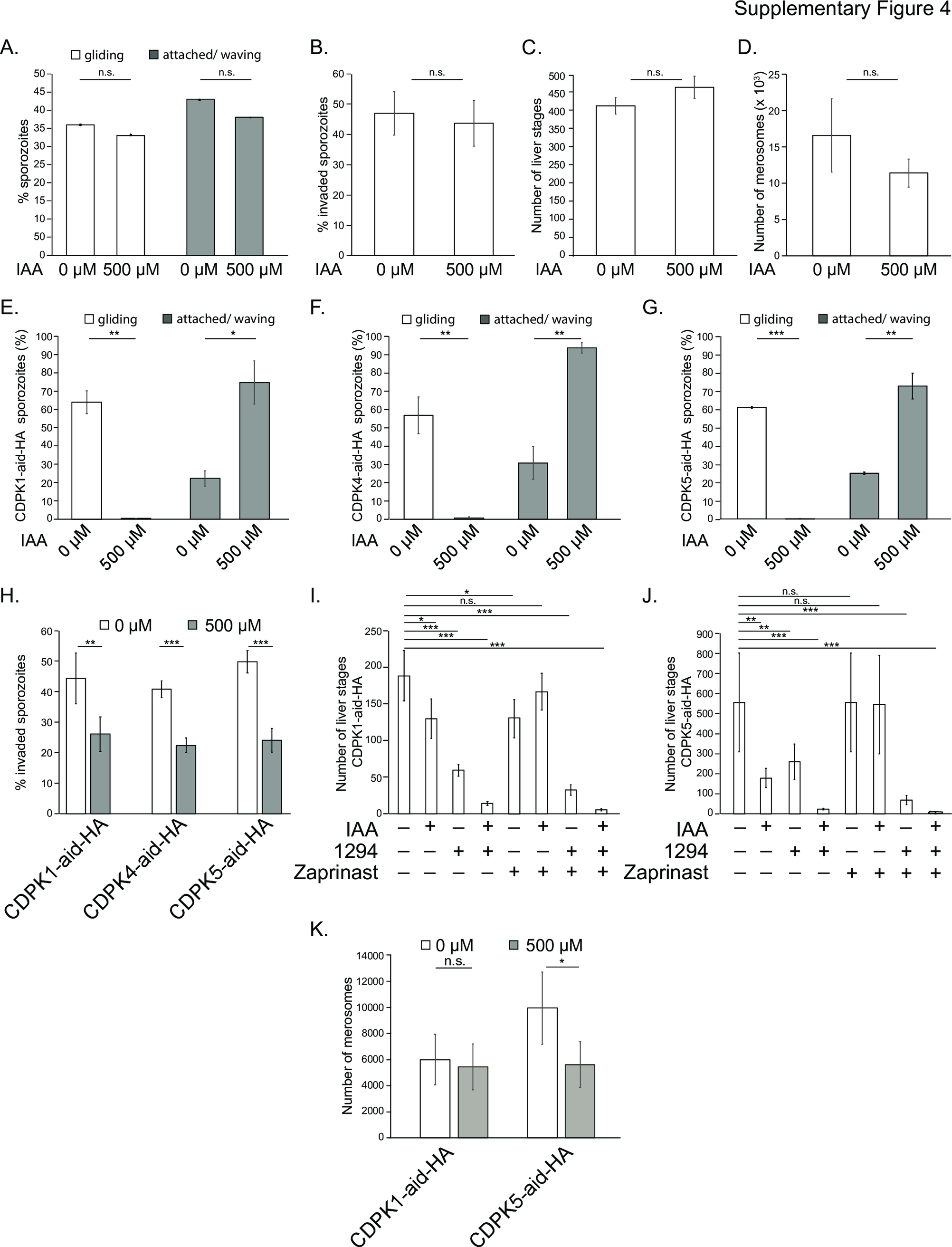

Supplement: S4 Fig — A-D) Effect of IAA treatment on gliding (A), invasion (B), liver-stage formation (C) and merosome formation (D) by Ostir1 control parasites. Results shown are the mean of 2–4 experiments with 3 technical replicates (± SD). E-G) Effect of IAA-treatment on gliding by CDPK1-aid-HA (E), CDPK4-aid-HA (F) and CDPK5-aid-HA (G) sporozoites. Results shown are the mean of 3–4 experiments (± SD). H) Effect of IAA-treatment on invasion by CDPK1-aid-HA, CDPK4-aid-HA and CDPK5-aid-HA sporozoites. Results shown are the mean of 3–4 experiments, each with 3–4 technical replicates (± SD). I) Effect of simultaneous depletion of CDPK1-aid-HA protein and inhibition of CDPK4 activity or enhancement of PKG activity. Results shown are the mean of 3 experiments, each with 3 technical replicates (± SD). J) Effect of simultaneous depletion of CDPK5-aid-HA protein and inhibition of CDPK4 activity or enhancement of PKG activity. Results shown are the mean of 3 experiments, each with 3 technical replicates (± SD). K) Effect of IAA-treatment on merosome formation by CDPK1-aid-HA and CDPK5-aid-HA liver stages. Results shown are the mean of 2 experiments with CDPK1-aid-HA and 3 experiments with CDPK5-aid-HA, each with 3 technical replicates (± SD). Data were analyzed using an unpaired t-test or one-way ANOVA, Dunnett’s multiple comparisons test, non-significant (n.s.) P > 0.05, * P value < 0.05, ** P value < 0.005, *** P value < 0.0005. (TIF) [file ppat.1008131.s012.tif]

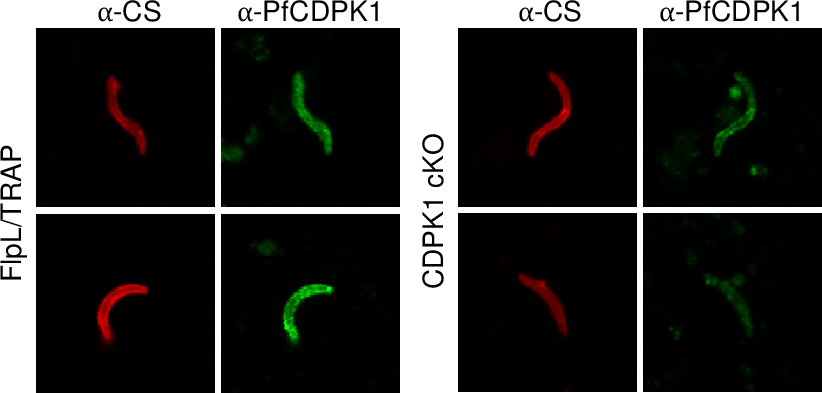

Supplement: S5 Fig — CDPK1 cKO sporozoites were generated through FlpL-mediated deletion of the CDPK1 ORF in FlpL-expressing parasites (FlpL/TRAP). PbCDPK1 was detected using an anti-PfCDPK1 antibody. Anti-CS was used as control. Images shown are representative of CDPK1 expression in these sporozoites. (TIF) [file ppat.1008131.s013.tif]

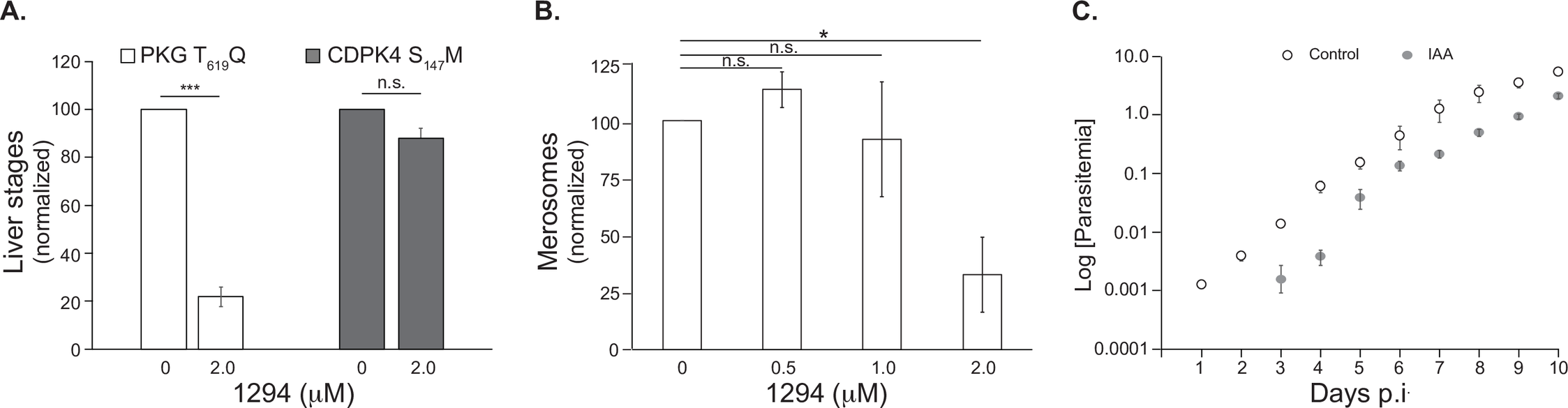

Supplement: S6 Fig — A) Specificity of 1294 in sporozoites was interrogated by testing its effects on HepG2 infection by ‘gatekeeper’ mutants of PKG (T619Q) and CDPK4 (S147M). Liver stages were quantified at 48 h p.i. The number of liver stages formed under each condition was normalized to vehicle-treated controls. Results shown for CDPK4 S147M are average (± SEM) of 3 experiments, each with 3–4 technical replicates. Results shown for PbPKG T619Q are average (± SD) of one experiment with 4 technical replicates. B) Dose-dependent inhibition of merosome formation by 1294. Merosomes and detached cells were quantified at 65–68 h p.i. with PbLuc sporozoites. Compound was added to infected HepG2 cultures at 48 h p.i. and refreshed every 12 h. The number of merosomes/detached cells (± SEM) formed in each condition was normalized to vehicle-treated controls. The experiment was performed twice with technical triplicates. C) Growth rate of CDPK5-aid-HA parasites in erythrocytes. Parasitemias (± SD) of mice infected with vehicle- or IAA-treated CDPK5-aid-HA merosomes were determined daily. Data are from a representative experiment (5 mice/group). Data were analyzed using an unpaired t-test, non-significant (n.s.) P > 0.05, * P value < 0.05, ** P value < 0.005, *** P value < 0.0005. (TIF) [file ppat.1008131.s014.tif]

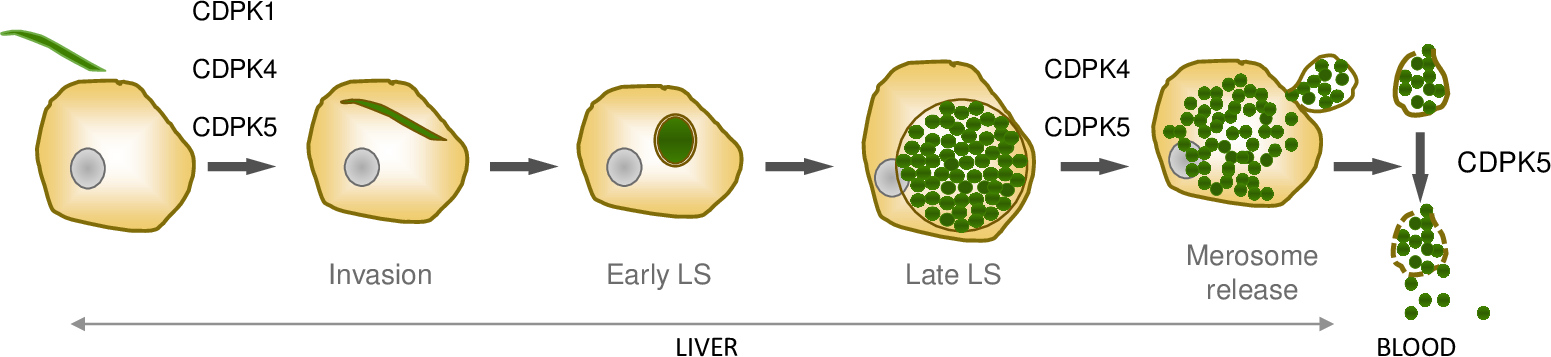

Supplement: S7 Fig — CDPK family members play a critical role during several steps of hepatocyte infection. Sporozoite motility and consequently infection of hepatocytes requires CDPK1, 4 and 5. In addition, CDPK4 and 5 function in the formation or release of merosomes from the infected hepatocyte. The release of hepatic merozoites from merosomes requires CDPK5. (TIF) [file ppat.1008131.s015.tif]
